# Midgut Microbial Community of *Culex quinquefasciatus* Mosquito Populations from India

**DOI:** 10.1371/journal.pone.0080453

**Published:** 2013-11-29

**Authors:** Kshitij Chandel, Murlidhar J. Mendki, Rasesh Y. Parikh, Girish Kulkarni, Sachin N. Tikar, Devanathan Sukumaran, Shri Prakash, Brahma D. Parashar, Yogesh S. Shouche, Vijay Veer

**Affiliations:** 1 Vector Management Division, Defence Research and Development Establishment, Jhansi Road, Gwalior, India; 2 National Centre for Cell Sciences, Pune University Campus, Pune, Maharashtra, India; Metabiota, United States of America

## Abstract

The mosquito *Culex quinquefasciatus* is a ubiquitous species that serves as a major vector for west nile virus and lymphatic filariasis. Ingestion of bloodmeal by females triggers a series of physiological processes in the midgut and also exposes them to infection by these pathogens. The bacteria normally harbored in the midgut are known to influence physiology and can also alter the response to various pathogens. The midgut bacteria in female *Cx. quinquefasciatus* mosquitoes collected over a large geographical area from India was studied. Examination of 16S ribosomal DNA amplicons from culturable microflora revealed the presence of 83 bacterial species belonging to 31 bacterial genera. All of these species belong to three phyla i.e. Proteobacteria, Firmicutes and Actinobacteria. Phylum Proteobacteria was the most dominant phylum (37 species), followed by Firmicutes (33 species) and Actinobacteria (13 species). Phylum Proteobacteria, was dominated by members of γ-proteobacteria class. The genus *Staphylococcus* was the largest genus represented by 11 species whereas *Enterobacter* was the most prevalent genus and recovered from all the field stations except Leh. Highest bacterial prevalence was observed from Bhuj (22 species) followed by Nagrota (18 species), Masimpur (18 species) and Hathigarh (16 species). Whereas, least species were observed from Leh (8 species). It has been observed that individual mosquito harbor extremely diverse gut bacteria and have very small overlap bacterial taxa in their gut. This variation in midgut microbiota may be one of the factors responsible for variation in disease transmission rates or vector competence within mosquito population. The present data strongly encourage further investigations to verify the potential role of the detected bacteria in mosquito for the transmission of lymphatic filariasis and west nile virus. To the best of our knowledge this is the first study on midgut microbiota of wild *Cx. quinquefasciatus* from over a large geographical area.

## Introduction

Among the disease transmitting insects, the mosquitoes are the primary hosts for transmission of diseases like malaria, dengue, chikungunya, lymphatic filariasis, yellow fever etc., which together are responsible for several million deaths and hundreds of millions of cases every year. *Culex quinquefasciatus* Say is an important vector of west nile virus and filarial nematode, *Wuchereria bancrofti*. The later causes lymphatic filariasis in humans and presently over 120 million peoples are infected with filarial worm[1]. Isolation of Japanese encephalitis virus was also reported from field collected *Cx. quinquefasciatus* mosquitoes. [2].

Lymphatic filariasis is a major public health problem in India and Worldwide, it is estimated that 1.3 billion people from 83 countries are living at the risk of infection; however in India LF is endemic in 17 states and six union territories, and is responsible for one third of the global disease burden with about 554.2 million people at risk of infection, with 31 million parasite carriers and 23 million cases of symptomatic filariasis[1].

The bacteria colonizing midguts of insect vectors have drawn special attention for their interaction with both the insect hosts and pathogenic organisms[3]. Little is known about the midgut microflora of *Culex* mosquitoes and very few studies has been conducted to study the midgut microbiota of *Culex* mosquitoes [4], [5]. A comprehensive study has been conducted by us on the diversity of microbiota in the midgut lumen of *Cx. quinquefasciatus* in order to ascertain their potential role in disease transmission and for their exploitation in vector management. In the present study, wild populations of *Cx. quinquefasciatus* were collected from different locations from India to investigate geographical variation in their midgut bacterial community.

## Materials and Methods

### Sample collection

**Figure 1:**
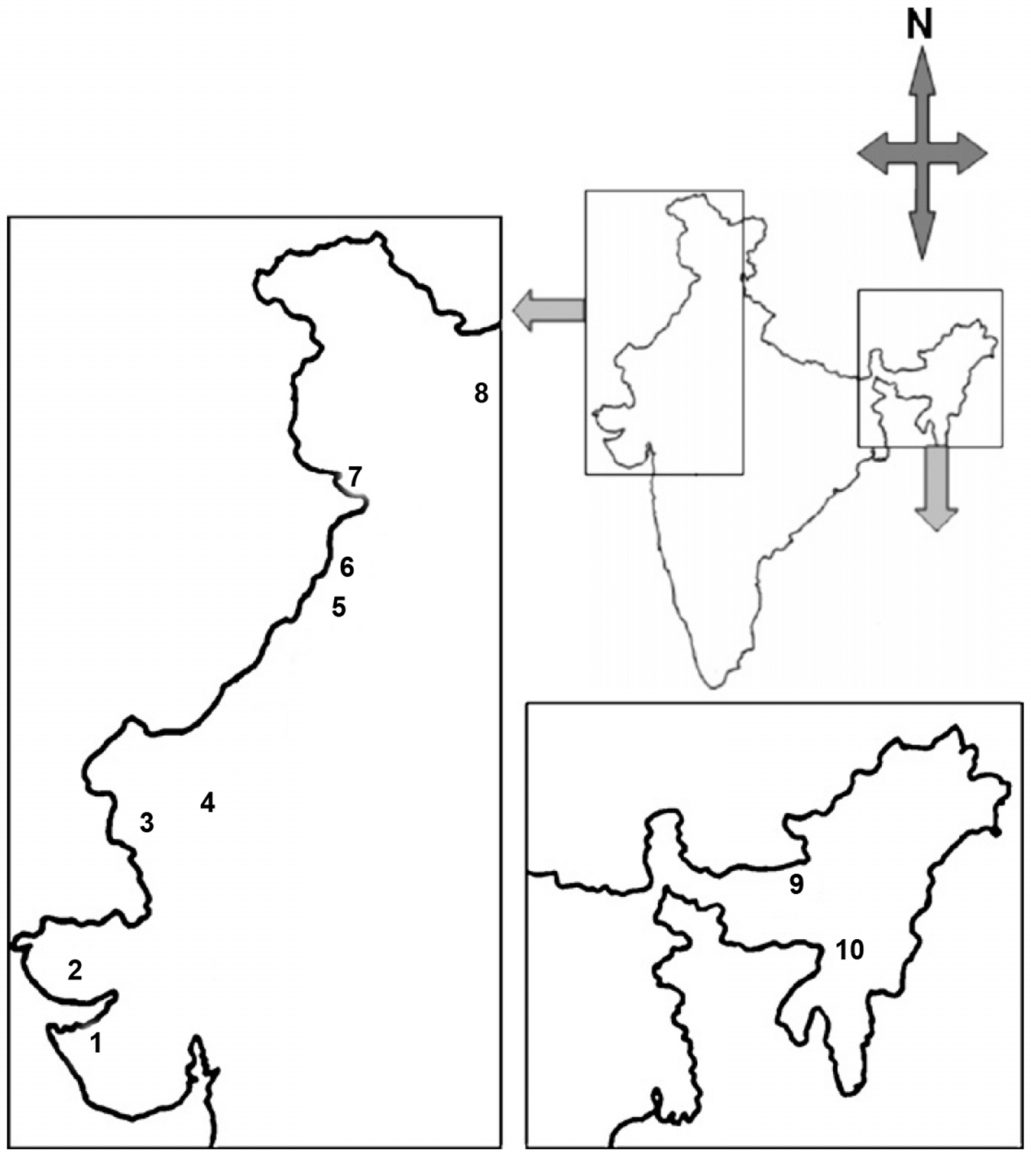
Collection sites of *Culex quinquefasciatus* mosquito in India. 1: Jamnagar(JMN), 2: Bhuj(BHJ), 3: Barmer(BAR), 4: Jodhpur(JOD), 5: Bathinda(BHT), 6: Amritsar(ASR), 7: Nagrota(NAG), 8: Leh(LEH), 9: Hathigarh(HGR), 10: Masimpur(MSM).

Study locations comprised 10 field stations situated in different climatic zones viz. coastal, arid, semi-arid, mountainous and subtropical (Fig. 1). Mosquitoes were collected from army cantonments from these field stations, with the permission of Director General Armed Forces Medical Services (DGAFMS), Ministry of Defence, Government of India. From each station, indoor resting adult *Cx. quinquefasciatus* females were collected from different sites at dawn and dusk. Collected mosquitoes were kept in pre-sterilized cages. Mosquitoes were anesthetized with chloroform and species was identified morphologically using standard taxonomic key. Adult mosquitoes were surface sterilized with 70% ethanol for 5 min followed by washing in phosphate buffered saline (PBS) (twice) before dissection for further processing of midgut isolates and bacterial cultivation according to Pidiyar *et. al.* [4]. Midguts were microscopically dissected out under sterile conditions and transferred individually to 100 µl of brain heart infusion broth (BHI broth) in 1.5 ml microcentrifuge tubes and enriched for 4 hrs at room temperature. After enrichment, an equal volume of 40% glycerol was added in each tube and stored in liquid nitrogen and transported to the laboratory for further study. From each station, 100 mosquitoes were dissected out and midgut samples were collected for bacterial diversity. To confirm the sterility of the procedure, two controls were also taken from each station which contained PBS from a mosquito's second wash.

### Isolation of bacterial flora

Midgut contents were ten-fold serially diluted up to 10^−8^ dilution in PBS and 100 µl of each dilution was spread on tryptose soya agar (TSA) supplemented with 5% sheep blood and incubated at 30°C for 48–72Hrs. The resulting bacterial colonies were grouped based on their colony morphology. Bacterial colonies that were morphologically distinct were selected and subcultured on TSA plates until a pure culture was obtained for further analysis.

### DNA extraction and PCR amplification of 16S rDNA

Pure bacterial isolates from mosquito midguts were subcultured in 5 ml tryptose soya broth (TSB) at 30°C for 24 hrs. Cell pellets were suspended in distilled water and lysed using repeated cycles of freezing and thawing, lysozyme and proteinase K treatment. DNA was isolated by phenol∶chloroform∶isoamyl alcohol (24∶25∶1) extraction and isopropanol precipitation protocol[6]. Complete 16S rRNA gene (Approx. 1.5 kb size) was amplified from extracted DNA of isolates as described by Pidiyar *et al*., [4] using eubacteria specific primers 27F 5′– CCA GAG TTT GAT CMT GGC TCA G – 3′ and 1525 R 5′ –TTC TGC AGT CTA GAA GGA GGT GWT CCA GCC-3′. Amplification of the 16S rRNA gene was confirmed by gel electrophoresis using 1% agarose. PCR products were purified using PEG-NaCl precipitation[6] and then sequenced on an ABI 3730 (Applied Biosystems Inc. Foster City, CA) automated DNA sequencer using the Big Dye termination kit at the National Centre for Cell Sciences (NCCS), Pune, India. For sequencing, three internal primers were used for generating large overlapping sequences. These sequences were then assembled into full length amplicons for analysis. Sequences were submitted to GenBank under the accession numbers **JN644482 to JN644627**.

### Sequence analysis

To identify the closest related sequence, obtained sequences were analyzed at the EzTaxon server (http://147.47.212.35:8080)[7]. For phylogenetic analysis, related sequences were retrieved from the database and multiple sequence alignment of sequences was carried out using the CLUSTAL W program available at http://www.ebi.ac.uk/Tools/msa/clustalw2/. Unaligned sequences at the beginning and end of the alignment file were trimmed using DAMBE Ver. 5.2.9 (http:/www.dambe.bio.uottawa.ca) and the alignment file was converted to MEGA format. Phylogenetic trees were constructed using >1200 base pair aligned sequences by the neighbor joining method using Kimura 2 distances parameter in MEGA 4.0 package[8]. One thousand bootstrap replicates were generated, and a consensus tree was derived.

### Statistical analysis

To study the species richness and diversity the following diversity indices were estimated: Simpson Index [9], Shannon Index, Evenness and Sφrrensen Index [10]. Good's coverage was calculated using following formula[11]: “Percent coverage  = [1−(n/N)]×100” Where n = number of bacterial species represented by single isolate and N =  is the total number of bacterial isolates from particular location.

## Results

Microbial diversity in the midgut of the *Cx. quinquefasciatus* mosquito was analyzed for the presence of aerobic bacteria. Adult female *Cx. quinquefasciatus* mosquitoes were collected during the post monsoon season where mosquito population density was quite high.

To study aerobic bacterial diversity, we used a 16S rRNA gene sequence based approach. Characterization of gut microbes in *Cx. quinquefasciatus* collected from all 10 locations using a culture dependent method led to the identification of 31 bacterial genera, including 83 species from 3 phyla. The 16S rDNA sequence of bacterial isolates were aligned with reference strains available in GenBank and used for construction of phylogenetic trees as shown in figures S1–S3 (supplementary material), revealed the relatedness among the bacteria identified. The most prominent phylum was Proteobacteria with the greatest number of species isolated from mosquito midguts and included 37 bacterial species (45%) from 15 genera. Firmicutes was the second largest phylum with 33 bacterial species (40%) from 9 genera. The least frequently isolated phylum was Actinobacteria where only 13 (15%) species from 7 genera were identified.

Bhuj belongs to a coastal climatic zone. In this region, 65 samples (65%) of mosquito midguts were found to be positive for bacterial growth and 22 bacterial species from 14 genera were reported from this region (Table 1). Of the 22 bacterial species, 50% (11 species) belong to phylum Proteobacteria and 41% (9 species) and 9% (2 species) belong to phyla Firmicutes and Actinobacteria, respectively (Table 2). *Bacillus thuringiensis* was the most frequently isolated bacterial species and isolated from 36% of the samples analyzed (Table 2). Four species viz., *Bacillus nealsonii, Staphylococcus caprae, Acinetobacter baumannii* and *Pantoea anthophila* were isolated only once. Percent coverage of bacterial diversity from this region was 97.6% (Table 1).

**Table 1:** showing occurrence of bacterial taxa identified, Good's coverage and diversity indices at 10 different field stations.

	Sample location									
	BHJ	JMN	JOD	BAR	ASR	BHT	NAG	LEH	HGR	MSM
**Number of mosquito midgut analyzed**	100	100	100	100	100	100	100	100	100	100
**Midgut positive for bacterial growth**	65	69	25	22	25	32	67	20	85	82
**Total taxa identified**	22	12	13	11	13	15	18	8	16	18
**Total bacterial isolates recovered (N)**	168	115	44	28	30	43	139	30	187	203
**Bacterial species represented by single isolate (n)**	4	3	2	4	5	4	2	2	3	2
Good's coverage [(1−n/N)*100]	97.62	97.39	95.45	85.71	83.33	90.70	98.56	93.33	98.40	99.01
**Simpson diversity Index**	0.900	0.873	0.897	0.869	0.898	0.905	0.908	0.813	0.903	0.903
**Shannon diversity Index**	2.615	2.186	2.393	2.197	2.405	2.518	2.57	1.832	2.47	2.528
**Evenness**	0.622	0.741	0.842	0.818	0.852	0.827	0.72	0.781	0.74	0.696

**Table 2:** of bacterial species identified from *Cx. quinquefasciatus* midgut and their occurrence in each location with their sequence accession number.

S. No.	Species Identified	Bacterial prevalence(Out of 100 mosquitoes midgut)									
		BHJ	JMN	JOD	BAR	ASR	BHT	NAG	LEH	HGR	MSM
1	*Acinetobacter baumannii*	1 (JN644493)			2 (JN644533)						
2	*Acinetobacter beijerinckii*	2 (JN644494)									1 (JN644620)
3	*Acinetobacter junii*							14 (JN644576)			
4	*Acinetobacter lwoffii*	5 (JN644495)						13 (JN644577			
5	*Acinetobacter pittii*										12 (JN644621)
6	*Acinetobacter radioresistens*								2 (JN644591)		
7	*Acinetobacter schindleri*							7 (JN644578)		1 (JN644599)	3 (JN644622)
8	*Acinetobacter soli*		19 (JN644513)							12 (JN644600)	
9	*Aerococcus urinaeequi*							1 (JN644571)			
10	*Aeromonas enteropelogenes*									1 (JN644602)	
11	*Aeromonas hydrophila*							9 (JN644579)		31 (JN644601)	
12	*Aeromonas ichthiosmia*					3 (JN644542)					
13	*Aeromonas veronii*						4 (JN644562)				
14	*Arthrobacter creatinolyticus*	3 (JN644482)	1 (JN644504)								
15	*Bacillus anthracis*						1 (JN644555)				
16	*Bacillus aryabhattai*					2 (JN644540)					
17	*Bacillus cereus*	5 (JN644484)									
18	*Bacillus circulans*						1 (JN644554)				
19	*Bacillus flexus*			4 (JN644518)							
20	*Bacillus licheniformis*										3 (JN644612)
21	*Bacillus nealsonii*	1 (JN644485)					4 (JN644556)				
22	*Bacillus safensis*						2 (JN644557)	2 (JN644572)			
23	*Bacillus subtilis*	9 (JN644487)	15 (JN644507)			1 (JN644541)		22 (JN644573)			11 (JN644613)
24	*Bacillus thuringiensis*	36 (JN644486)									
25	*Citrobacter braakii*							9 (JN644584)			
26	*Citrobacter freundii*						3 (JN644567)				
27	*Delftia lacustris*									6 (JN644603)	
28	*Enterobacter asburiae*		9 (JN644515)			1 (JN644549)	2 (JN644564)				
29	*Enterobacter cancerogenus*	3 (JN644497)					5 (JN644565)	3 (JN644583)		2 (JN644607)	7 (JN644618)
30	*Enterobacter cloacae*	19 (JN644498)	17 (JN644514)	6 (JN644526)	3 (JN644534)	4 (JN644548)	8 (JN644566)			16 (JN644608)	15 (JN644619)
31	*Enterobacter ludwigii*	7 (JN644496)				2 (JN644550)				15 (JN644609)	
32	*Enterococcus caccae*					2 (JN644546)					
33	*Enterococcus faecalis*	15 (JN644488)	21 (JN644508)			4 (JN644545)				19 (JN644596)	22 (JN644614)
34	*Enterococcus hirae*		9 (JN644509)								
35	*Enterococcus silesiacus*					1 (JN644547)	2 (JN644558)				
36	*Escherichia coli*					3 (JN644544)		5 (JN644580)		24 (JN644604)	
37	*Escherichia hermannii*					1 (JN644551)					
38	*Exiguobacterium aurantiacum*							6 (JN644574)			
39	*Exiguobacterium indicum*			2 (JN644520)	2 (JN644531)						
40	*Exiguobacterium profundum*	2 (JN644489)	1 (JN644510)	1 (JN644519)	1 (JN644532)						
41	*Janibacter melonis*							2 (JN644568)			
42	*Klebsiella oxytoca*			2 (JN644527)	1 (JN644535)						
43	*Klebsiella pneumoniae*				4 (JN644536)			19 (JN644581)		22 (JN644605)	31 (JN644624)
44	*Klebsiella variicola*	3 (JN644499)									
45	*Kocuria carniphila*			2 (JN644516)							8 (JN644610)
46	*Kocuria marina*								1 (JN644586)	11 (JN644594)	
47	*Kocuria palustris*	5 (JN644483)									
48	*Kytococcus schroeteri*							1 (JN644569)			
49	*Lactococcus lactis*										6 (JN644615)
50	*Leucobacter tardus*									7 (JN644595)	10 (JN644615)
51	*Lysinibacillus macroides*			1 (JN644521)				2 (JN644575)		1 (JN644597)	2 (JN644616)
52	*Microbacterium arborescens*		12 (JN644505)								
53	*Microbacterium imperiale*		1 (JN644506)								
54	*Microbacterium maritypicum*							2 (JN644570)			
55	*Microbacterium oxydans*			6 (JN644517)	5 (JN644529)			7 (JN644585)			
56	*Micrococcus lylae*				1 (JN644530)						
57	*Micrococcus yunnanensis*						2 (JN644553)				
58	*Morganella morganii*				6 (JN644537)						
59	*Pantoea anthophila*	1(JN644500)									
60	*Pantoea dispersa*					5 (JN644543)					
61	*Proteus vulgaris*				2 (JN644538)						
62	*Providencia alcalifaciens*				1 (JN644539)		1 (JN644563)				
63	*Providencia rettgeri*	2 (JN644501)									14 (JN644625)
64	*Pseudomonas beteli*										2 (JN644627)
65	*Pseudomonas cuatrocienegasensis*								1 (JN644592)		
66	*Pseudomonas protegens*								3 (JN644593)		
67	*Pseudomonas stutzeri*			7 (JN644528)				15 (JN644582)		10 (JN644606)	21 (JN644626)
68	*Serratia marcescens*	21 (JN644503)									
69	*Shigella flexneri*					1 (JN644552					1 (JN644623)
70	*Sporosarcina luteola*						3 (JN644559				
71	*Staphylococcus agnetis*	9 (JN644491)	6 (JN644511)								
72	*Staphylococcus arlettae*								7 (JN644587)		
73	*Staphylococcus caprae*	1 (JN644490)									
74	*Staphylococcus epidermidis*			2 (JN644522)					8 (JN644588)		
75	*Staphylococcus gallinarum*			4 (JN644523)							
76	*Staphylococcus haemolyticus*						1 (JN644560		2 (JN644589)		
77	*Staphylococcus hominis*						4 (JN644561)				
78	*Staphylococcus saprophyticus*									9 (JN644598)	34 (JN644617)
79	*Staphylococcus succinus*			5 (JN644525)							
80	*Staphylococcus warneri*								6 (JN644590)		
81	*Staphylococcus xylosus*			2 (JN644524)							
82	*Stenotrophomonas maltophilia*	12 (JN644502)									
83	*Vagococcus fluvialis*	6 (JN644492)	4 (JN644512)								

Second location from coastal region is Jamnagar, from where 69 (out of 100) samples were found to be positive for bacterial growth (Table 1) and 12 bacterial species from 9 genera and 3 phyla were isolated. In Jamnagar, Firmicutes was the most dominating phylum with 6 species (belonging to 5 genera) accounting for 50% of the total species identified. Two and three species from 2 genera belonged to phyla Proteobacteria and Actinobacteria respectively (Table 2). Most frequently isolated species was *Enterococcus faecalis* which was recorded in 21% of the samples analyzed, followed by *Acinetobacter soli* which was isolated from 19% of the samples analyzed and *Enterobacter cloacae* which was isolated from 17% of the samples. *Arthrobacter creatinolyticus, Microbacterium imperiale* and *Exiguobacterium profundum* were represented by single individual (Table 2). Similar to Bhuj the percent coverage from Jamnagar was 97.4% (Table 1).

From arid climatic zone two stations namely Jodhpur and Barmer situated in thar desert were selected for study. From Jodhpur, only 25% of the samples were found to be positive for bacteria and 13 species (belonging to 9 genera) from 3 phyla were identified (Table 1). Firmicutes was the most prevalent phylum, represented by 62% of the total bacterial species identified. Three species (23%) from 3 genera belong to phylum Proteobacteria and the remaining 2 species (15%) from 2 genera belong to phylum Actinobacteria. The most frequently isolated species was *Pseudomonas stutzeri* which is isolated from 7% of the total samples analyzed (Table 2), and in Jodhpur the percent coverage was 95.5% (Table 1).

In Barmer region, percent positivity of mosquito midguts for bacteria was only 22%. Among these, a total of 11 species (belonging to 9 genera) were identified (Table 1). Phylum Proteobacteria was the largest phylum represented by 7 species (64%) belonging to 6 genera. Two species with 2 genera belonged to Firmicutes and 2 species from 1 genus belonged to phylum Actinobacteria (Table 2). The most frequently isolated species was *Morganella morganii*, that was isolated from 6% of the total samples analyzed followed by *Microbacterium oxydans* which was recovered from 5% of the samples. The percent coverage in Barmer region was calculated as 85.7% (Table 1).

Amritsar is a semiarid climatic zone showed a 25% positivity of mosquito midgut for presence of bacteria. A total of 13 species belonging to 8 genera and 2 phyla were identified (Table 1). Phylum Proteobacteria was the most dominant phylum, wherein 8 of 13 species (72%) belonged to this phylum. The remaining 5 species (38%) belonged to phylum Firmicutes (Table 2). *Pantoea dispersa* was the most frequently isolated bacterial species and was isolated from 5% of the samples. Five species, *Bacillus subtilis, Enterococcus silesiacus, Enterobacter asburiae, Escherichia hermannii* and *Shigella flexneri* were isolated only once. The Good's coverage from Amritsar was quite low and calculated 83.3% (Table 1).

In Bathinda, thirty two midguts (32%) were found to be positive for bacterial flora. A total of 15 species from 9 genera were identified from this region (Table 1). The most prevalent phylum was Firmicutes, followed by Proteobacteria, and then Actinobacteria. Eight species (53%) belonged to Firmicutes, six species (40%) belonged to Proteobacteria, and 1 species (7%) belonged to phylum Actinobacteria. *Enterobacter cloacae was* the most frequently isolated species and was isolated from 8% of the total samples. In this region, *Bacillus circulans, Bacillus thuringiensis, Staphylococcus haemolyticus* and *Providencia alcalifaciens* were represented by single isolate (Table 2). In comparison to Amritsar, the percent coverage from Bathinda was quite high and calculated as 90.1% (Table 1).

The mountainous climatic zones namely Nagrota and Leh were studied. In Nagrota, the percent positivity was 67%, with eighteen species (belonging to 14 genera) isolated (Table 1). In Nagrota, 50% of total species identified belonged to phylum Proteobacteria, the most dominant phylum. Five species from 4 genera belonged to phylum Firmicutes, and 4 species from 3 genera belonged to phylum Actinobacteria (Table 2). *Bacillus subtilis* was the most frequently isolated species and was isolated from 22% of the mosquito midguts analyzed. Two species, *Kytococcus schroeteri* and *Aerococcus urinaeequi* were represented by only one isolate. Percent coverage for bacterial diversity from Nagrota was 98.6% (Table 1).

Another field station from the mountainous climatic zone was Leh. From Leh only 20 mosquito midguts were found to be positive for bacteria and 8 species (belonging to 4 genera) from 3 phyla were isolated (Table 1). Phylum Firmicutes was the biggest phylum with 4 species (50%), followed by phylum Proteobacteria where only 3 species were isolated, and 1 species belonged to phylum Actinobacteria. Out of these, *Staphylococcus epidermidis*, was the most frequently isolated bacterium isolated from 8% of the total samples analyzed. *Kocuria marina* and *Pseudomonas cuatrocienegasensis* were represented by only one isolate(Table 2). The percent coverage of the bacterial diversity from Leh was 93.3% (Table 1).

Hathigarh and Masimpur represent the tropical climate of Assam. Eighty five percent of the samples from Hathigarh were found to be positive for bacteria and 16 species (belonging to 14 genera) were identified (Table 1). Phylum Proteobacteria was the most dominant phylum with 11 species (69%) from 7 genera represented. Three species (19%) from 3 genera belong to phylum Firmicutes. The least frequently isolated phylum in this region was Actinobacteria with 2 species (12%) belonging to 2 genera (Table 2). In Hathigarh, *Aeromonas hydrophila* was the most frequently isolated species identified in 31% of the total samples analyzed followed by *Escherichia coli* and *Klebsiella pneumoniae* which were recovered from 24 and 22% of the mosquito population. Three species viz., *Lysinibacillus macroides, Acinetobacter schindleri* and *Aeromonas enteropelogenes* were represented by single isolates. Bacterial diversity from Hathigarh was quite rich as Good's percent coverage was recorded 98.4% (Table 1).

The percent positivity was 82% with 18 species (belonging to 14 genera) from 2 phyla isolated from Masimpur (Table 1). In this region, 56% of the isolated bacterial species (10 species from 6 genera) belonged to phylum Proteobacteria, 33% (6 species from 5 genera) belonged to phylum Firmicutes and the remaining 11% (2 species from 2 genera) belonged to phylum Actinobacteria (Table 2). The most frequently isolated species was *Staphylococcus saprophyticus* which was isolated from 34% of the total samples studied followed by *Klebsiella pneumoniae* which was recovered from 31% samples. The least frequently isolated species were *Acinetobacter beijerinckii* and *Shigella flexneri* which were represented by single isolates. The Good's coverage at Masimpur was 99% (Table 1).

Simpson richness index, Shannon diversity index and Evenness diversity indices were estimated from all the locations and are summarized in Table 1. The values of Simpson diversity index ranged from 0.813 to 0.908, the lowest value was estimated for Leh and the highest value was for Nagrota region. Shannon diversity index is another widely used index for comparing diversity between various habitats [12]. In the present study, values of Shannon (H) index ranged from 1.832 to 2.615 (Table 1) with the highest value for Bhuj and lowest value for the Leh region. Evenness was used for estimating how well the species are evenly distributed among the individuals. Values of evenness were between 0.622 – 0.852 (Table 1). The highest evenness was recorded for Amritsar and the least was estimated for the Bhuj region. Sφrrenson coefficient is also very widely used similarity index. Pair wise matrix of Sφrrenson index and number of species shared by two populations are shown in Table 3. The values of Sφrrenson index ranged from 0 to 0.529 with the highest value calculated between Masimpur and Hathigarh. The lowest values of Sφrrenson index (0) were calculated between the communities where none of the species was shared.

**Table 3:** Pair wise matrix of common species (Upper half) and Sφrrensen similarity index (Lower half).

	BHJ	JMN	JOD	BAR	ASR	BHT	NAG	LEH	HGR	MSM
**BHJ**	-	7	2	3	4	4	3	0	4	6
**JMN**	0.412	-	2	2	4	2	1	0	3	3
**JOD**	0.114	0.160	-	5	1	1	3	1	3	4
**BAR**	0.182	0.174	0.417	-	1	2	2	0	2	2
**ASR**	0.229	0.320	0.077	0.033	-	3	2	0	4	4
**BHT**	0.216	0.148	0.066	0.207	0.214	-	2	1	2	2
**NAG**	0.150	0.066	0.194	0.138	0.129	0.121	-	0	7	6
**LEH**	0	0	0.095	0	0	0.870	0	-	1	0
**HGR**	0.211	0.214	0.207	0.148	0.276	0.129	0.412	0.083	-	**9**
**MSM**	0.300	0.20	0.258	0.222	0.258	0.121	0.333	0	**0.529**	-

## Discussion

The bacteria inhabiting mosquito midgut has drawn special attention in recent past due to their interactions with both mosquito host as well as disease causing parasites. The present work generated detailed information about aerobic bacterial flora in the midgut lumen of field caught *Cx. quinquefasciatus* female mosquitoes. Richness and diversity of microbes associated with the field collected adult mosquitoes was found to be quite high in all the samples.

Midgut contents from any of the field stations have not shown presence of anaerobic bacteria or fungi (data not shown). Though *Aspergillus* sp. has been isolated from *Anopheles stephensi* larval gut but adult mosquitoes did not harbour any fungus[13]. Absence of fungus in adult gut indicates that the environment of midgut lumen of the adult mosquito is not conducive for the fungi [14]. Similarly anaerobic bacteria were also not recovered from midgut content from any of the field stations.

Conversely, a large number of aerobic bacteria were isolated from the *Culex* midgut contents from all the field stations. Our result demonstrates that the aerobic microbial flora of the adult mosquito midgut is a complex one and is dominated by gram negative gammaproteobacteria. The maximum number of the bacterial species was identified from Bhuj followed by Nagrota, Masimpur and Hathigarh. The least number of bacterial species were isolated from Leh region (8 species belonging to 4 genera). The climate of Leh is not favorable for mosquito breeding and mosquitoes can only breed in the short period where the temperature regime is suitable for their breeding. Mosquitoes mainly acquire bacteria either from their larval habitat or from the environment during the process of nectar feeding or blood feeding. Because of very low temperatures, environmental bacterial load is also very low which was also reflected in mosquito midgut bacterial diversity. High numbers of bacterial species were recorded from the areas where rainfall and RH was high such as Bhuj, Nagrota, Hathigarh and Masimpur . Though the rainfall and high humidity stimulates the bacterial growth in environment, in the present study no significant correlation was observed with rainfall, relative humidity and temperature. Since many biotic and abiotic factors account for the distribution of microbes in environment, it is possible that other unidentified and ecologically important factors, or interactions between these factors may account for the differences in richness observed across populations.

The extent of prevalence of culturable bacteria in different species of mosquitoes and in different population of the same species seems to be quite variable. In present study percent positivity for presence of bacteria ranged from 20% to 85% and maximum incidence was recorded in *Cx. quinquefasciatus* population from Hathigarh whereas least was found in Leh region. Most of the species were unevenly distributed among the host population. Overall bacterial prevalence is less than 50%. Similarly Lindh et. al. (2005) reported bacterial prevalence of culturable bacteria among 15% of the mosquito population[15]. However, Djadid et. al. (2011) observed very high bacterial prevalence in field collected *An. stephensi* from Iran but could not retrieve any culturable bacteria from midgut of *Anopheles maculipennis* [16]. It can be inferred from present study that either possibly only a part of mosquito population acquires bacteria or large number of bacteria are not stable midgut residents. Alternatively, it could also be possible that mosquitoes harbouring bacteria were not culturable under the culture conditions provided in present study.

The diversity index quantifies diversity in a community and describes its numerical structure. Simpson gave the probability of any two individuals drawn at random from an infinitely large community belonging to different species [9]. In the present study, the values of Simpson index ranged from 0.813 to 0.908 with the highest and the lowest between Nagrota and Leh, respectively. The value of Simpson index increases with diversity. The Shannon (H) index is another widely used index for comparing diversity between various habitats[12]. In the present study, the values of Shannon index ranged between 1.832 to 2.615 being the highest for Bhuj and lowest from the Leh region. Generally the values of Shannon index ranged between 1.5 to 3.5, where values above 3 indicates that the diversity of the habitat is rich and stable, whereas values under 1.0 indicate that there is pollution and degradation of habitat structure and diversity is not stable. The Shannon index is a representation of both species abundance and evenness, when either of these two factors increases, the diversity index also increases. The Shannon index has maximum values when all the species are equally abundant in community. Evenness was used for the estimating how well the species are evenly distributed among the individuals. The highest evenness was recorded for Amritsar station indicating that bacterial species at Amritsar are evenly distributed among the individuals as compared to other field stations. The lowest evenness was recorded from Bhuj region indicating that species are less evenly distributed and some species more dominant than the others. The Sφrrenson coefficient is a very widely used similarity index. This mainly depends upon the number of species common in two populations analyzed. Values of the Sφrrenson Index vary between 0 to 1 where 0 indicates none of the species is shared by two communities, whereas a value of 1 indicates that both the communities having exactly the same bacterial composition. Values of Sφrrenson index ranged from 0 to 0.529 in the present study. The maximum numbers of common species were found between Masimpur and Hathigarh, which resulted in the highest Sφrrenson value. Nine bacterial species were shared by these two stations and the value of Sφrrenson index is 0.529. Conversely, none of the species were shared between LEH-BHJ, LEH-JMN, LEH-BAR, LEH-ASR, LEH-NAG and LEH-MSM, hence the values of Sφrrenson index was also 0 for these pairs of stations.

There were a large number of soil and environmental bacteria isolated in this study, such as species of *Acinetobacter, Microbacterium, Micrococcus, Stenotrophomonas* and *Bacillus* sp., and these isolates showed regional variation. This suggests that the local soil and water environment plays an important role in colonization of the mosquito midgut with regional bacteria encountered at breeding sites or during nectar or blood feeding.

Most of the genera recovered in present study have already been reported from midgut of various mosquito vector species. Of the 31 bacterial genera 23 has been previously reported to inhabit mosquito gut. However, 8 genera *Arthrobacter, Janibacter, Kytococcus, Leucobacter, Aerococcus, Sporosarcina, Vagococcus* and *Delftia* are the first report as midgut microflora of *Culex* mosquito. Members of genera *Acinetobacter*, *Aeromonas*, *Bacillus, Enterobacter*, *Enterococcus, Klebsiella*, *Pantoea*, *Pseudomonas*, *Serratia*, *Staphylococcus* and *Stenotrophomonas*, have been frequently reported from mosquito gut in previous studies and our results are consistent with those of the earlier reports[4], [5], [15], [17]–[28]. This suggests that at least a fraction of mosquito midgut inhabitants could be common for different mosquito species inhabiting similar environments and may represent evolutionary conservation of association between bacteria and mosquito gut. Comparative analysis of bacterial diversity from adult *Culex* mosquito revealed the high prevalence of genus *Enterobacter* (14.4%), *Bacillus* (11.9%), *Staphylococcus* (10.0%), *Enterococcus* (9.5%), *Acinetobacter* (9.4%), *Klebsiella* (8.2%), *Pseudomonas* (5.9%) and *Aeromonas* (4.8%) in present study. Bacterial isolates belong to genus *Enterobacter* were recovered from all the field locations except Leh and comprise of a major part of midgut microbiota of *Culex* mosquitoes in the present study which is in accordance with earlier reports [4], [5], [18], [29]. It has been reported previously that species of *Enterobacter* are the most common bacteria isolated from insect gut[30].

The midgut bacterial infection in wild mosquito populations may influence parasite transmission and could contribute to understanding variation in vectorial capacity observed by same species in different locations because naturally existing microorganism in mosquito midgut have important roles to determine parasite survival and development. Mosquitoes are known to respond to infection by disease causing pathogen and elicit a specific immune response against them [31]. Same immune response gene are also expressed in response to midgut bacteria and this raises the possibility that the presence of specific bacteria in the midgut may alter the vectorial efficiency at which a pathogen is transmitted by a vector mosquito [24]. Thus, the midgut bacterial composition has a considerable effect on the survival of pathogens in the midgut environment. Earlier studies have indicated that the susceptibility of *Cx. quinquefasciatus* mosquito for JE virus increases when *Pseudomonas sp*. and *Acinetobacter sp*. were incorporated in the mosquito blood meal [32]. Similarly, in a study on occurrence of *Klebsiella sp.* and *Pseudomonas sp.* in mosquito midguts reported that mosquitoes with *Pseudomonas* in their midgut showed a higher prevalence of malaria sporozoites, whereas females infected with *Klebsiella sp.* could not support parasite development [33], [34]. *Enterobacter* isolated from wild mosquito populations from Zambia inhibits midgut epithelium invasion of *Plasmodium falciparum* through generation of reactive oxygen species (ROS) in *Anopheles* mosquitoes and resulted in inhibition of parasite development in mosquito gut [35]. Similarly, improved conversion of oocystes from ookinets was observed when mosquito fed with plasmodium infected blood containing antibodies raised against midgut bacteria[36].

The large numbers of bacteria present in the vector midgut are capable of producing factors that can kill parasites. Haemolysin produced by *Enterobacter cloacae, Serratia marcescens, Escherichia coli, Enterococcus faecalis* exerts activity against both prokaryotic and eukaryotic cells[3]. Other molecules produced by *Serratia marcescens* are protease and prodigiosin. Prodigiosin has been shown to have potent activity against *Trypenosoma curzi* and derivatives of prodigiosin also have shown marked activity against *Plasmodium falciparum* [3].

To the best of our knowledge this is the first attempt at comparative cataloguing of midgut microbiota of *Cx. quinquefasciatus* mosquitoes over a large geographical area in India. Identification and characterization of mosquito midgut flora is likely to contribute towards better understanding of mosquito biology including longevity, reproduction and mosquito microbe interaction that may be important to develop novel strategies for vector control. We embarked on this microbiological survey to identify bacteria that might be used in future efforts to develop paratransgenesis as a mechanism to block vectorial transmission by mosquito. Riehle et al., have mentioned essential requirement for suitable candidate for paratransgenesis[37] and some bacteria isolated in present study could be a suitable candidate for screening for paratransgenesis.

## Supporting Information

Figure S1 **Dendrogram showing phylogenetic affiiliation of bacterial isolates belonging to phylum Actinobacteria.** The tree was constructed using neighbor joining algorithm with Kimura 2 parameter distances. Number at the nodes indicate percent bootstrap values (1000 replicates). The bar indicates the Jukes-Cantor evolutionary distance.
(TIF)

Figure S2 **Dendrogram showing phylogenetic affiiliation of bacterial isolates belonging to phylum Firmicutes.** The tree was constructed using neighbor joining algorithm with Kimura 2 parameter distances. Number at the nodes indicate percent bootstrap values (1000 replicates). The bar indicates the Jukes-Cantor evolutionary distance.
(TIF)

Figure S3 **Dendrogram showing phylogenetic affiiliation of bacterial isolates belonging to phylum Proteobacteria.** The tree was constructed using neighbor joining algorithm with Kimura 2 parameter distances. Number at the nodes indicate percent bootstrap values (1000 replicates). The bar indicates the Jukes-Cantor evolutionary distance.
(TIF)
