# Supplementary figures and images for: Midgut Microbial Community of Culex quinquefasciatus Mosquito Populations from India
Source: PLoS One. 2013 Nov 29;8(11):e80453. doi: 10.1371/journal.pone.0080453 (PMC3843677; doi:10.1371/journal.pone.0080453)

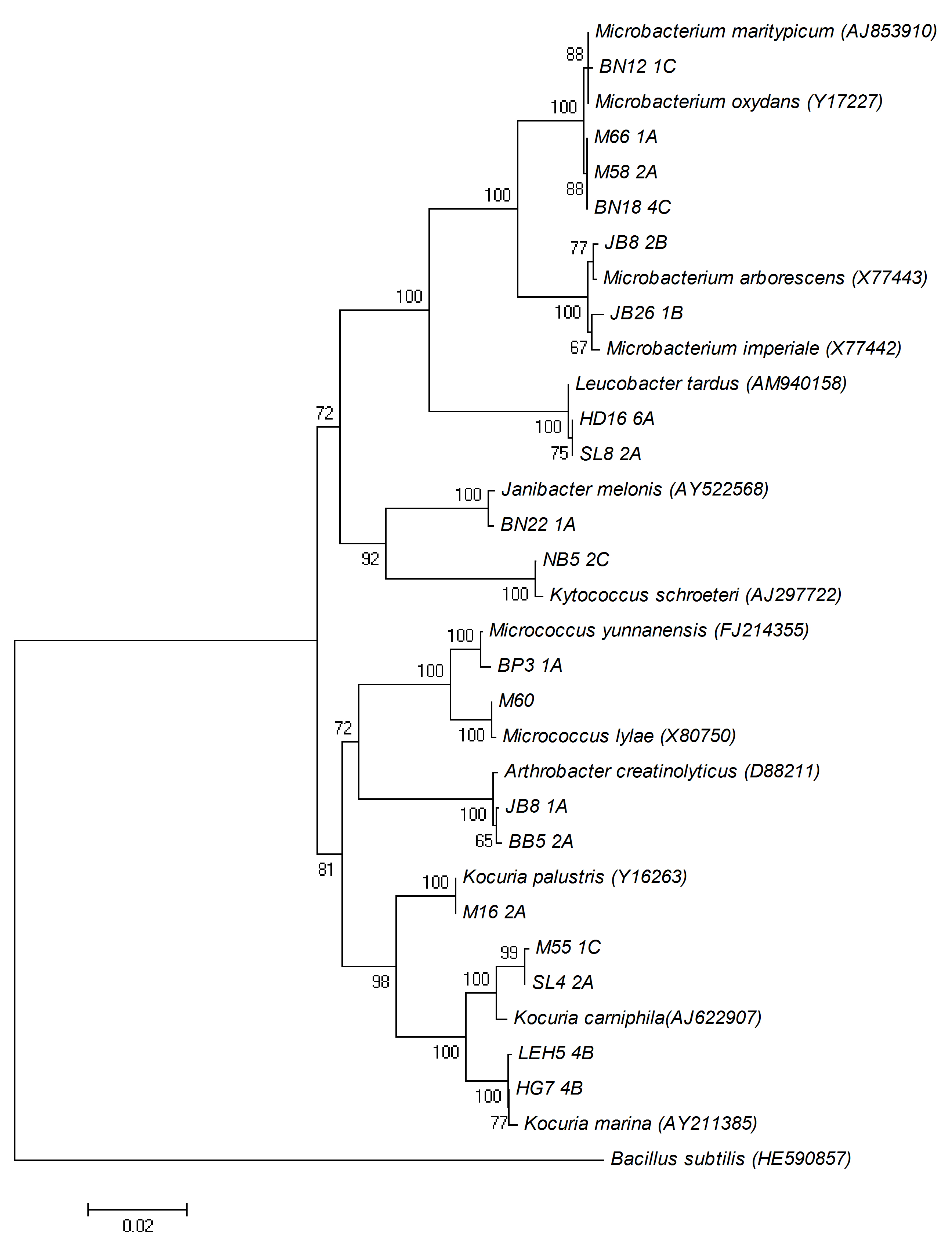

Supplement: Figure S1 — Dendrogram showing phylogenetic affiiliation of bacterial isolates belonging to phylum Actinobacteria. The tree was constructed using neighbor joining algorithm with Kimura 2 parameter distances. Number at the nodes indicate percent bootstrap values (1000 replicates). The bar indicates the Jukes-Cantor evolutionary distance. (TIF) [file pone.0080453.s001.tif]

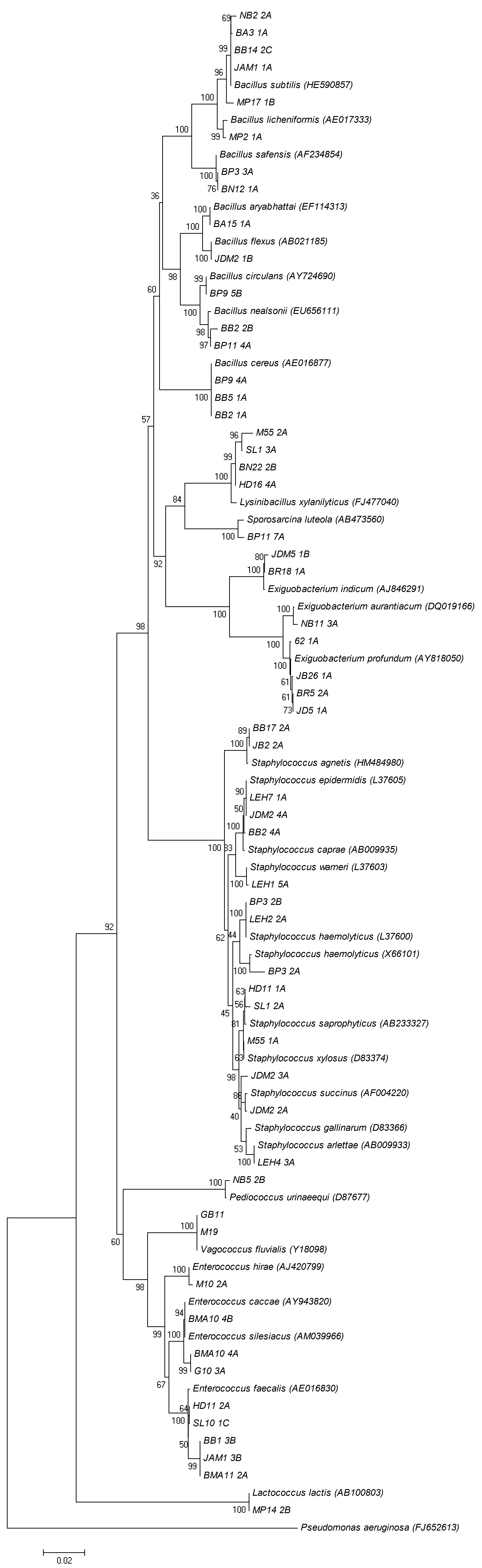

Supplement: Figure S2 — Dendrogram showing phylogenetic affiiliation of bacterial isolates belonging to phylum Firmicutes. The tree was constructed using neighbor joining algorithm with Kimura 2 parameter distances. Number at the nodes indicate percent bootstrap values (1000 replicates). The bar indicates the Jukes-Cantor evolutionary distance. (TIF) [file pone.0080453.s002.tif]

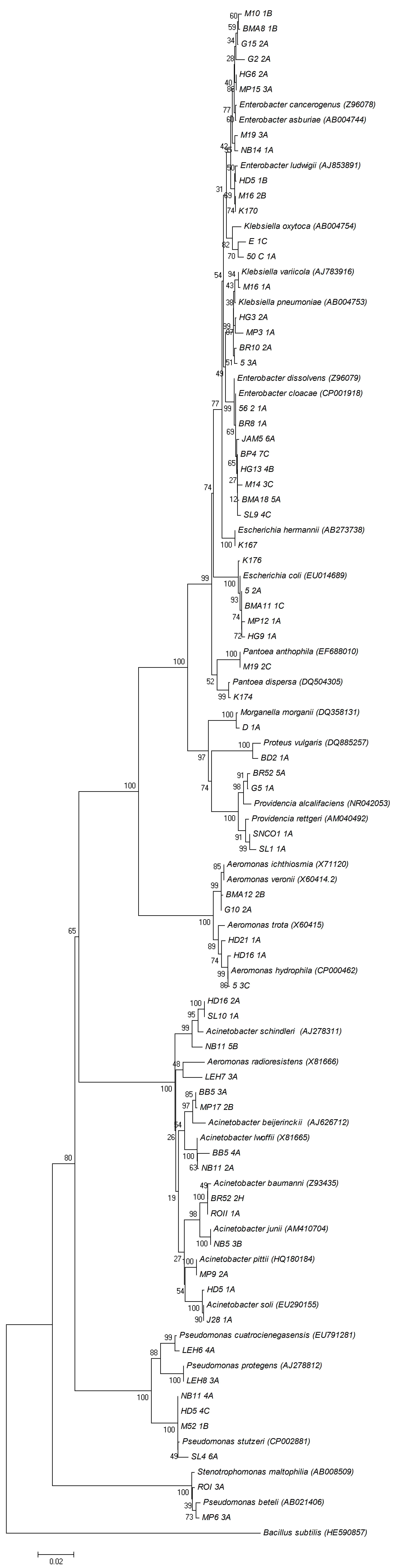

Supplement: Figure S3 — Dendrogram showing phylogenetic affiiliation of bacterial isolates belonging to phylum Proteobacteria. The tree was constructed using neighbor joining algorithm with Kimura 2 parameter distances. Number at the nodes indicate percent bootstrap values (1000 replicates). The bar indicates the Jukes-Cantor evolutionary distance. (TIF) [file pone.0080453.s003.tif]
